# Daidai Flower Essential Oil as a Multifunctional Natural Agent: Chemical Profile, Antimicrobial Activity, Preliminary Insecticidal Potential, and Improved Microbiological Quality of Fresh Arugula During Refrigerated Storage

**DOI:** 10.1111/1750-3841.71413

**Published:** 2026-08-25

**Authors:** Minhang Qiao, Joel Horacio Elizondo‐Luevano, Anis Ben Hsouna, Rania Ben Saad, Zhaojun Ban, Li Li, Jian Lou, Stefania Garzoli, Miroslava Kačániová

**Affiliations:** ^1^ Institute of Horticulture, Faculty of Horticulture and Landscape Engineering Slovak University of Agriculture Nitra Slovakia; ^2^ Laboratory of Natural Sciences, Biomolecular Innovation Group, Faculty of Agronomy Universidad Autónoma de Nuevo León Ciudad General Escobedo Nuevo León Mexico; ^3^ Laboratory of Biotechnology and Plant Improvement Centre of Biotechnology of Sfax Sfax Tunisia; ^4^ Department of Environmental Sciences and Nutrition, Higher Institute of Applied Sciences and Technology of Mahdia University of Monastir Monastir Tunisia; ^5^ School of Biological and Chemical Engineering, Zhejiang University of Science and Technology, Zhejiang Provincial Key Laboratory of Chemical and Biological Processing Technology of Farm Products Zhejiang Provincial Collaborative Innovation Center of Agricultural Biological Resources Biochemical Manufacturing Hangzhou China; ^6^ Key Laboratory for Agro‐Products Postharvest Handling of Ministry of Agriculture and Rural Affairs, College of Biosystems Engineering and Food Science Zhejiang University Hangzhou China; ^7^ Department of Chemistry and Technologies of Drug Sapienza University Rome Italy; ^8^ School of Medical & Health Sciences VIZJA University Warszawa Poland

**Keywords:** antimicrobial activity, arugula, *Callosobruchus maculatus*, *Citrus aurantium* var. *daidai*, linalool, MALDI‐TOF MS, natural preservatives

## Abstract

This study evaluates the potential of daidai flower essential oil (DFEO) (*Citrus aurantium* var. *daidai*) as a natural preservative for fresh leafy vegetables. The chemical composition of DFEO was determined by gas chromatography coupled with mass spectrometry (GC–MS), revealing a neroli‐type profile dominated by linalool (42.8%), limonene (19.9%), and linalyl acetate (15.3%). The tested batch of DFEO exhibited broad‐spectrum antimicrobial activity, with Gram‐positive bacteria showing the highest susceptibility. In situ experiments in model food systems confirmed concentration‐dependent antimicrobial effects, with inhibition values reaching up to 89.47%. The application of DFEO in combination with vacuum packaging significantly improved the microbiological quality of fresh arugula during refrigerated storage (4°C, 14 days), reducing coliform bacteria by 1.26 log CFU/g compared with untreated controls. Matrix‐assisted laser desorption/ionization time‐of‐flight mass spectrometry (MALDI‐TOF MS) analysis of culture‐dependent bacterial isolates revealed a dominance of *Pseudomonas* spp., which are key contributors to spoilage of leafy vegetables. Although DFEO also demonstrated insecticidal activity against stored‐product pests, its primary relevance lies in its antimicrobial efficacy in food systems. These findings demonstrate that DFEO has potential as a natural preservative for fresh produce, particularly when combined with vacuum packaging, contributing to improved microbiological quality of minimally processed vegetables during refrigerated storage.

## 1 Introduction

Arugula (*Eruca sativa* Mill., family Brassicaceae) is one of the fastest‐growing leafy vegetables and is characterized by its distinctive aromatic flavor, which is largely attributed to the presence of glucosinolates and their degradation products primarily isothiocyanates. In addition to its nutritional value, including vitamins A and C, flavonoids, carotenoids, and minerals, arugula has been associated with several health‐promoting effects, including anticarcinogenic, hepatoprotective, and diuretic properties (Bell and Wagstaff 2019). Despite its increasing consumption, arugula remains one of the less extensively studied leafy vegetables from a microbiological perspective. Previous studies have shown that the phyllosphere microbiota of arugula is dominated by members of the family Enterobacteriaceae, and the presence of genes conferring resistance to clinically relevant antibiotics has also been reported, representing a potential concern from a food safety standpoint (Cernava et al. 2019). Since arugula is predominantly consumed raw, it is particularly susceptible to microbial contamination, which highlights the need for effective strategies aimed at extending its shelf life (Inestroza‐Lizardo et al. 2016).

In recent years, the identification of microorganisms in foods and food‐processing environments has undergone a significant transformation due to the introduction of matrix‐assisted laser desorption/ionization time‐of‐flight mass spectrometry (MALDI‐TOF MS). This method has proven to be faster, more accurate, and more cost‐effective than conventional phenotypic and molecular identification techniques (Becker and Lupetti 2023). Its application in food microbiology includes the identification of foodborne pathogens, spoilage microorganisms, as well as phytopathogenic bacteria associated with the surfaces of fresh vegetables (Surányi et al. 2023). Most previous studies investigating the microbiota of fresh vegetables rely on 16S rRNA gene sequencing, which typically provides identification primarily at the genus level because this genomic region lacks sufficient discriminatory power to reliably distinguish closely related species within genera such as *Pseudomonas* or *Pantoea*. The Bruker MALDI Biotyper system enables rapid and reliable identification of isolates based on the comparison of protein fingerprint spectra with a reference database, where a score ≥ 2.000 corresponds to reliable species‐level identification; however, this culture‐dependent approach identifies only morphologically selected, culturable isolates and does not capture the full viable or total microbial community present in a sample, and should therefore be regarded as complementary to, rather than a replacement for, sequencing‐based approaches (Becker and Lupetti 2023; Surányi et al. 2023).

Plant essential oils are complex mixtures of volatile compounds with a wide range of biological activities. Their antimicrobial activity is primarily associated with terpenoid components that disrupt cell membrane integrity, interfere with cellular metabolism, and ultimately lead to cell death (Hou et al. 2022). Daidai (*Citrus aurantium* var. *daidai* Tanaka, family Rutaceae), a Japanese cultivar of bitter orange, produces a floral essential oil closely related to neroli oil, typically rich in linalool and linalyl acetate as dominant constituents, with a substantial proportion of limonene, β‐pinene, and α‐terpineol (Liang et al. 2021). Linalool (3,7‐dimethyl‐1,6‐octadien‐3‐ol) is an acyclic monoterpene alcohol with well‐documented antimicrobial activity against both Gram‐negative and Gram‐positive bacteria, including *Pseudomonas fluorescens*, *P. aeruginosa*, *Listeria monocytogenes*, and *Staphylococcus aureus*. Based on published data for its major constituents, the antimicrobial activity of daidai flower essential oil (DFEO) is attributed to disruption of the cell membrane, alteration of membrane potential, and inhibition of enzymes involved in cellular energy metabolism, as reported for linalool in previous studies (Gao et al. 2019; Guo et al. 2021; Liu et al. 2020). Limonene, another quantitatively important component of plant essential oils, has demonstrated insecticidal activity against stored‐product pests, including *Callosobruchus maculatus*, and has also been identified as a potential oviposition deterrent for this species (Barbosa et al. 2021; Hudaib et al. 2010). The simultaneous evaluation of antimicrobial and insecticidal activities within a single study is therefore justified by the shared chemical basis of both effects, as the dominant compounds of DFEO linalool and limonene are well documented for both types of bioactivity, enabling a more comprehensive characterization of the biological potential of this essential oil.

Stored‐product pests such as *C. maculatus* (Fabricius, 1775) and *Megabruchidius dorsalis* (Fahraeus, 1839) (Coleoptera: Chrysomelidae) cause significant losses of stored legumes and cereals in tropical and subtropical regions. The increasing resistance of these pests to synthetic insecticides, together with the environmental and health risks associated with their use, has stimulated growing interest in botanical alternatives based on plant essential oils. Their insecticidal activity is primarily attributed to mechanisms involving acetylcholinesterase inhibition and the modulation of octopaminergic receptors (Isman 2020; Kalpna et al. 2022).

Although the microbiota of fresh leafy vegetables has been characterized in several studies using 16S rRNA gene sequencing, available data on the microbiota of arugula during storage remain limited. Most published studies focus on the overall composition of microbial communities, while less attention has been paid to the culturable microbiota and its dynamics during postharvest storage. The combination of culture‐based approaches with MALDI‐TOF MS identification therefore allows a more detailed characterization of the specific cultivable bacterial isolates associated with arugula and enables the monitoring of changes in the culture‐dependent isolate profile during storage and following the application of natural antimicrobial agents such as essential oils.

Although the antimicrobial and insecticidal activities of citrus essential oils, linalool‐rich oils, limonene, and linalyl acetate have been widely reported in the literature, the novelty of the present study lies in (i) the application of DFEO to fresh arugula, a leafy vegetable for which no previous essential oil preservation study has been published; (ii) the combination of DFEO with vacuum packaging as a combined preservation strategy; (iii) the use of MALDI‐TOF MS for species‐level identification of the culture‐dependent bacterial isolate profile of arugula during refrigerated storage; and (iv) the simultaneous evaluation of antimicrobial and insecticidal activities of DFEO within a single integrated study.

The aim of this study was to (i) determine the chemical composition of DFEO (*C. aurantium* var. *daidai*) using gas chromatography coupled with mass spectrometry (GC–MS) analysis; (ii) evaluate its antimicrobial activity using the disc diffusion method and microdilution assays (MIC_50_, MIC_90_) against reference bacterial and yeast strains; (iii) verify antimicrobial activity under in situ conditions using pear, peach, kohlrabi, and potato as model food matrices; (iv) assess the effect of the essential oil in combination with vacuum packaging on the microbial quality of fresh arugula during 14 days of storage at 4°C; (v) identify the microbiota isolated from arugula using MALDI‐TOF MS; and (vi) evaluate the insecticidal potential of DFEO against *C. maculatus* and *M. dorsalis* using a fumigation assay.

While the antimicrobial activity of DFEO was initially screened using model food matrices (pear, peach, kohlrabi, and potato) to simulate contaminated food surfaces under controlled conditions, fresh arugula represents the primary food matrix of this study, used in the storage experiment to evaluate the practical preservation potential of DFEO under real refrigerated storage conditions. The insecticidal activity was included to provide a comprehensive characterization of the multifunctional biological potential of DFEO as a natural bioactive agent.

## 2 Materials and Methods

### 2.1 Characterization of DFEO

DFEO (*C. aurantium* var. *daidai*, neroli type, batch no. 20240512) was used as the tested bioactive compound in this study. The essential oil was purchased from GuiLin Four Seasons Sunshine (Guilin, Guangxi, China). According to the supplier's certificate of quality, the essential oil is a clear light yellow to amber liquid with a characteristic fresh floral aroma, a relative density at 20°C ranging from 0.860 to 0.876, and a refractive index at 20°C between 1.4610 and 1.4740. The optical rotation at 20°C ranged from +2° to +15°, with an acid value ≤ 2.0 and an ester value of 26–65. The essential oil was stored in a dark glass vial at 4°C and protected from direct sunlight until further use. Only one commercial batch of DFEO was used in this study (batch no. 20240512). Since essential oil composition can vary depending on plant origin, harvest season, extraction method, and storage conditions, the results of this study reflect the biological activity of this specific batch and may not be fully representative of DFEO in general.

### 2.2 Chemical Composition of DFEO

The volatile profile of DFEO (*C. aurantium* var. *daidai* essential oil) was analyzed using GC–MS. Analyses were performed using a Clarus 500 instrument (PerkinElmer, Waltham, Massachusetts, USA) equipped with a flame ionization detector (FID) and coupled to a mass spectrometer. Separation of compounds was achieved on a capillary column Varian Factor Four VF‐5.

The oven temperature program was set as follows: an initial temperature of 60°C, followed by an increase at a rate of 6°C/min to 220°C, where it was maintained for 20 min. Helium was used as the carrier gas at a constant flow rate of 1 mL/min.

Compound identification was performed by comparing the obtained mass spectra with the Wiley 2.2 and NIST 11 spectral libraries. Linear retention indices (LRI) were calculated based on a homologous series of *n*‐alkanes (C_8_–C_25_) analyzed under identical conditions and subsequently compared with literature data.

The relative content of individual compounds was expressed as the percentage of peak area relative to the total chromatogram area. No internal standard or correction factor was used. All analyses were performed in triplicate. This approach provides semiquantitative data; the relative percentages may not accurately reflect the true mass concentrations of individual compounds.

### 2.3 Evaluation of Antimicrobial Activity

#### 2.3.1 Tested Microorganisms and Cultivation Conditions

The antimicrobial activity of DFEO was evaluated against selected reference bacterial and yeast strains. The bacterial panel included the Gram‐positive species *Enterococcus faecalis* (CCM 4224), *L. monocytogenes* (CCM 4699), and *S. aureus* (CCM 4423), as well as the Gram‐negative species *Escherichia coli* (CCM 3954), *Salmonella enterica* subsp. *enterica* (CCM 3807), and *Yersinia enterocolitica* (CCM 7204ᵀ). The yeast panel consisted of opportunistic species *Candida albicans* (CCM 8186), *Candida glabrata* (CCM 8270), *Candida krusei* (CCM 8271), *Candida parapsilosis* (CCM 8260), and *Candida tropicalis* (CCM 8223).

Bacteria were cultivated in Mueller–Hinton broth (MHB; Oxoid, Basingstoke, UK), whereas yeasts were cultivated in Sabouraud dextrose broth (SDB; Oxoid, UK). Incubation was carried out for 24 h at 37°C for bacteria and 25°C for yeasts. Prior to testing, microbial suspensions were adjusted to a turbidity corresponding to 0.5 McFarland standard, equivalent to approximately 1.5 × 10^8^ CFU/mL.

#### 2.3.2 Disc Diffusion Assay

The primary screening of antimicrobial activity of DFEO was performed using the disc diffusion method. Sterile filter paper discs (6 mm diameter) were impregnated with 10 µL of the tested essential oil and placed on the surface of Mueller–Hinton agar (MHA) inoculated with bacterial suspension or Sabouraud dextrose agar (SDA) inoculated with yeast suspension. After incubation for 24 h (37°C for bacteria and 25°C for yeasts), inhibition zones were measured in millimeters. Blank sterile filter paper discs (6 mm, Oxoid) without essential oil were used as negative controls. The following antimicrobial agents were used as positive controls: cefoxitin (30 µg/disc) for Gram‐positive bacteria, gentamicin (10 µg/disc) for Gram‐negative bacteria, and fluconazole (25 µg/disc) for yeasts (Oxoid, UK). All experiments were performed in triplicate.

#### 2.3.3 Determination of MIC_50_ and MIC_90_ Using the Microdilution Method

Minimum inhibitory concentrations (MIC_50_ and MIC_90_) of DFEO were determined using the broth microdilution method in sterile 96‐well microtiter plates. This resulted in a final inoculum density of approximately 7.5 × 10^7^ CFU/mL per well, selected to ensure a sufficiently strong and reproducible absorbance signal at 570 nm across the tested concentration range. Dilutions were prepared in MHB (for bacteria) and SDB (for yeasts). To ensure adequate dispersion of the hydrophobic essential oil in aqueous media, the stock solution was vortexed for 1 min prior to each dilution step. The tested concentration range was from 10 mg/mL to 0.00485 mg/mL. Negative controls contained only culture medium, while positive controls consisted of medium inoculated with microorganisms without the essential oil. Since DFEO was dispersed directly in the culture broth without an additional solvent or emulsifier, solvent‐interference controls were not required; potential interference of DFEO itself with absorbance was excluded using the DFEO‐only blank wells described below. Additionally, DFEO‐only blank wells containing broth with the corresponding concentration of essential oil without microbial inoculum were included to exclude potential absorbance interference of DFEO at 570 nm. After 24‐h incubation (37°C for bacteria and 25°C for yeasts), microbial growth was quantified spectrophotometrically by measuring absorbance at 570 nm using a Glomax microplate reader (Promega, USA). MIC_50_ and MIC_90_ values, representing the essential oil concentrations inhibiting microbial growth by 50% and 90%, respectively, relative to the untreated growth control, were calculated by probit regression analysis of the concentration–response data using JMP 18 (SAS Institute, Cary, North Carolina, USA). All experiments were performed in triplicate.

### 2.4 In Situ Evaluation of Antimicrobial Activity of DFEO on Food Matrices

To simulate food contamination, selected bacterial and yeast strains were applied to fresh plant matrices (pear, peach, kohlrabi, potato). The raw materials were washed, cut into slices with a uniform thickness of approximately 0.5 cm, and dried under sterile conditions.

Individual slices were transferred to sterile Petri dishes (60 mm) and inoculated with 100 µL of microbial suspensions adjusted to a standardized cell density of 0.5 McFarland standard (approximately 1.5 × 10^8^ CFU/mL). Filter paper discs impregnated with DFEO solutions prepared in ethyl acetate (concentrations 500, 250, 125, and 62.5 µg/L) were attached to the inner side of the Petri dish lids, ensuring exposure to the vapor phase. A volume of 100 µL of the respective solution was applied to each filter disc, corresponding to actual DFEO amounts of 0.007 µg (62.5 µg/L), 0.014 µg (125 µg/L), 0.028 µg (250 µg/L), and 0.056 µg (500 µg/L) per disc. Ethyl acetate was selected as the solvent due to its high volatility and negligible antimicrobial activity at the volumes applied, ensuring that observed inhibition could be attributed exclusively to the volatile components of DFEO. Control samples contained discs impregnated with ethyl acetate without the essential oil. The Petri dishes were hermetically sealed with parafilm and incubated for 7 days at 37°C for bacteria and 25°C for yeasts. The extent of microbial inhibition was evaluated semiquantitatively using ImageJ software (NIH, USA).

### 2.5 Storage Experiment With Arugula

Fresh arugula (*Eruca sativa* Mill.) was purchased from a commercial retail network on the day the experiment was initiated. The purchased arugula was originally packaged in a plastic bag; after opening, it was rinsed with distilled water and allowed to drain freely on a sterile stainless‐steel rack at laboratory temperature. The samples were not mechanically dried to avoid damage to the leaf tissue. Subsequently, the arugula was divided into portions of 25 g using sterile scissors.

The experiment was designed as a factorial design with two monitored factors: (i) vacuum packaging (yes/no) and (ii) essential oil application (yes/no). The samples were divided into four groups:

- C1 (−V/−EO)—without vacuum packaging and without essential oil application
- C2 (+V/−EO)—vacuum packaging without essential oil application
- C3 (−V/+EO)—essential oil application without vacuum packaging
- EO (+V/+EO)—essential oil application followed by vacuum packaging

For the treated groups (C3 and EO), 25 mg of DFEO was weighed and mixed with 250 µL of 0.5% Tween 80 solution; this mixture was applied to the surface of the 25‐g arugula samples, corresponding to a final applied dose of 1.0‐mg DFEO per gram of arugula sample. Tween 80 was used solely as an emulsifier to facilitate dispersion of the hydrophobic essential oil in aqueous solution. At the concentration used (0.5%), Tween 80 has been reported to exhibit no antimicrobial activity against foodborne bacteria (Hilbig et al. 2016) and a separate Tween 80‐only control group was therefore not included. The mixture was gently applied to the surface of the 25‐g arugula samples to avoid mechanical damage to the leaf tissue. Subsequently, the samples were carefully mixed for approximately 1 min under aseptic conditions to ensure uniform distribution of the essential oil.

Samples from Groups C2 and EO were vacuum packaged using a Concept vacuum sealer (Choceň, Czech Republic) into food‐grade vacuum bags. Samples from Groups C1 and C3 were placed into the same type of food‐grade vacuum bags without vacuum sealing, ensuring that the only difference between packaging groups was the presence or absence of vacuum.

All samples were stored at 4°C ± 1°C for 14 days. Microbiological analyses were performed on Day 0 (three samples) and after 1, 7, and 14 days of storage. At each sampling time point (1, 7, and 14 days), 12 samples were analyzed (four groups × three replicates). In total, 39 samples were processed during the experiment.

### 2.6 Microbiological Evaluation of Arugula

At the defined sampling times, 25 g of arugula from each sample were transferred into sterile stomacher bags and homogenized with 225 mL of peptone water, achieving a dilution ratio of 1:10. Homogenization was performed using a Stomacher device for 2 min. From the homogenate, 10‐fold serial dilutions were prepared. From the appropriate dilutions, 100‐µL aliquots were inoculated onto the surface of selective agar media. The total viable count (TVC) was determined on Plate Count Agar (PCA; Oxoid, Basingstoke, UK) with incubation at 30°C for 48–72 h. Coliform bacteria were determined on Violet Red Bile Lactose Agar (VRBL; Oxoid, Basingstoke, UK) with incubation at 37°C for 24–48 h. The results were expressed as log CFU/g of sample. TVCs and coliform bacteria were selected as microbiological quality indicators in accordance with ISO 4832 and ISO 4833, respectively, which are standard methods applicable to fresh vegetables.

### 2.7 Identification of Microorganisms Using MALDI‐TOF MS

Morphologically distinct colonies were selected from the culture media and subcultured to obtain pure cultures. This culture‐dependent approach provides a profile of cultivable bacterial isolates and does not represent the complete microbial community of arugula. Identification of isolates was performed using the MALDI‐TOF MS Biotyper system (Bruker Daltonics, Bremen, Germany). Protein extraction was performed using the ethanol–formic acid extraction method. Colonies were suspended in 300 µL of distilled water and centrifuged at 10,000 × *g* for 2 min. After removal of the supernatant, 900 µL of ethanol were added and the samples were centrifuged again. The pellet was dried at room temperature and subsequently resuspended in 30 µL of 70% formic acid and 30 µL of acetonitrile. A 1‐µL aliquot of the extract was spotted onto the MALDI target plate and, after drying, overlaid with 1 µL of HCCA matrix (α‐cyano‐4‐hydroxycinnamic acid). Spectra were analyzed according to the manufacturer's recommendations and compared with the reference database. Identification scores were interpreted as follows: ≥ 2.000 reliable species‐level identification; 1.700–1.999 probable genus‐level identification; < 1.700 unreliable identification.

### 2.8 Evaluation of Insecticidal Activity

The insecticidal potential of DFEO was evaluated against two stored‐product pest species: *C. maculatus* (Fabricius, 1775) and *M. dorsalis* (Fahraeus, 1839) (Coleoptera: Chrysomelidae). Groups of 100 adult individuals of each species were placed into sterile Petri dishes (90 mm diameter, approximately 95 mL volume). Filter paper discs placed inside the dishes were treated with 100 µL of essential oil at concentrations of 100%, 50%, 25%, 12.5%, 6.25%, and 3.125%. To ensure uniform dispersion, the essential oil was diluted in 0.1% polysorbate 80 solution (Tween 80; Sigma–Aldrich, St. Louis, Missouri, USA). Petri dishes were hermetically sealed with parafilm to minimize evaporation of volatile compounds and incubated at laboratory temperature for 24 h, ensuring exposure of insects to the volatile components of the essential oil in a closed system (fumigation model). Control groups were prepared using filter paper discs impregnated only with 0.1% Tween 80 solution without essential oil; no mortality was recorded in the control groups for either species (0%), and Abbott's correction was therefore not required. After the exposure period, the numbers of live and dead individuals were recorded. All experiments were performed in triplicate. Mortality percentage was calculated to evaluate the concentration‐dependent insecticidal efficacy of the essential oil against the tested insect species.

### 2.9 Determination of Lethal Concentrations

The median lethal concentration (LC_50_) and the concentration causing 90% mortality (LC_90_) were estimated using probit analysis based on the mortality data obtained from the fumigation assays. The LC_50_ and LC_90_ values were calculated together with their 95% confidence intervals.

### 2.10 Statistical Analysis

The statistical analysis was conducted using IBM SPSS Statistics 26 (IBM Corp., Armonk, New York, USA). Data are presented as mean ± standard deviation (SD), and all experiments were performed in triplicate. Data normality and homogeneity of variances were verified prior to analysis. A two‐way ANOVA was used to evaluate the main effects of treatment and storage time and their interaction, followed by Tukey's HSD test at a significance level of *p* ≤ 0.05. All triplicates represent independent biological replicates. Graphical representations were generated using GraphPad Prism 9 (GraphPad Software, San Diego, California, USA).

## 3 Results

### 3.1 Chemical Composition of DFEO

**FIGURE 1:**
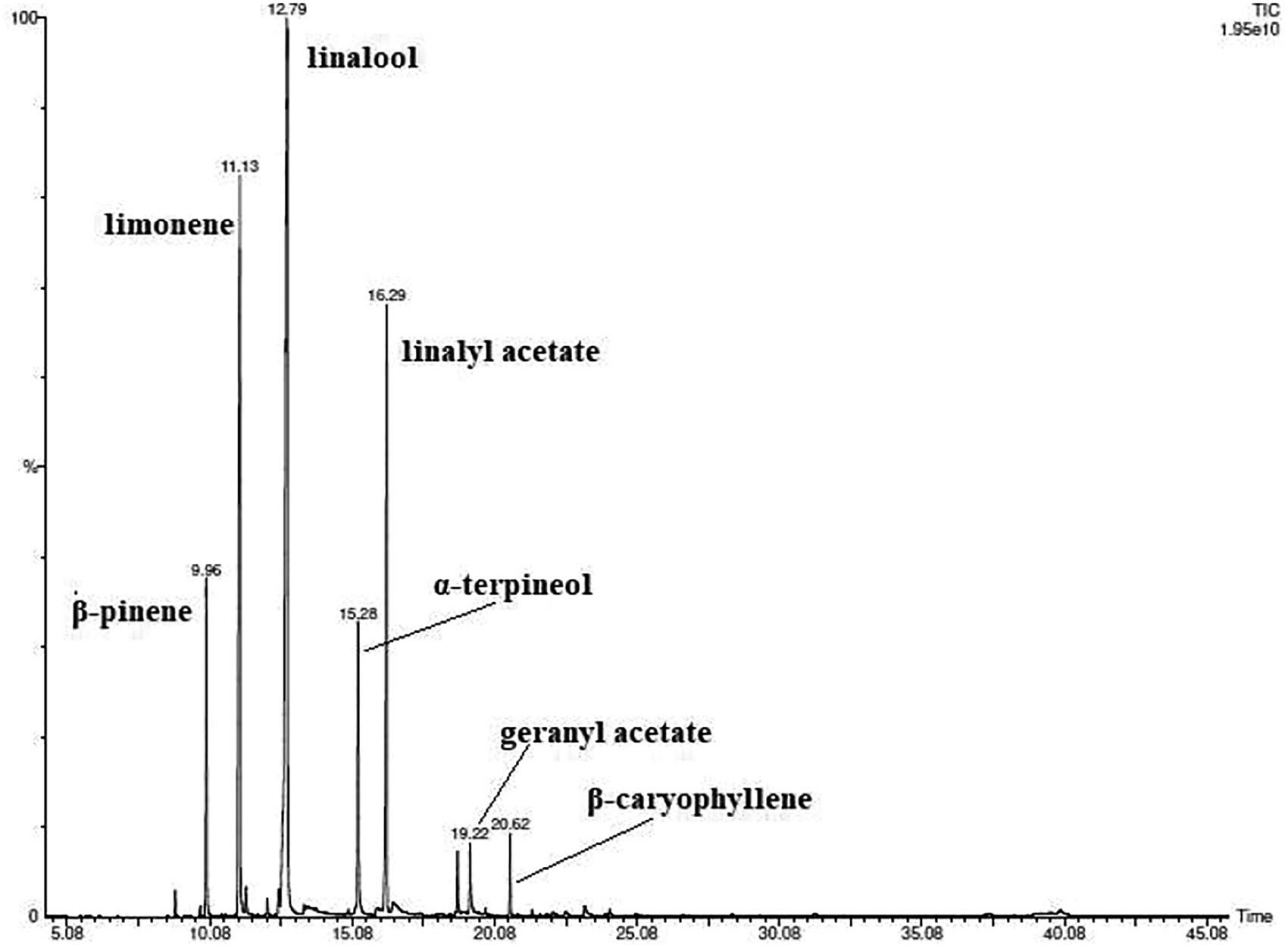
GC–MS chromatogram of DFEO.

GC–MS analysis of DFEO identified 21 major components representing 99.9% of the total essential oil composition. The oil exhibited a typical neroli‐type chemotype characterized by the predominance of oxygenated monoterpenes over monoterpene hydrocarbons (Table 1). The most abundant compound was linalool (42.8%), followed by limonene (19.9%) and linalyl acetate (15.3%). These three major constituents accounted for 78% of the total essential oil composition (Figure 1). Other notable components included α‐terpineol (6.7%) and β‐pinene (6.8%). Oxygenated monoterpenes constituted the dominant fraction with a total proportion of 51.5%; when monoterpenoids (ester derivatives of monoterpene alcohols, 18.3%) are included, the combined oxygenated fraction reached 69.8%. Linalool‐derived compounds (linalool + linalyl acetate + linalool oxides) accounted for 58.9% of the composition. Monoterpene hydrocarbons represented 27.3%, with limonene and β‐pinene as the predominant compounds. Sesquiterpene compounds were present only in minor amounts (2.1%), with β‐caryophyllene being the most abundant representative (1.3%). The chemical profile confirmed the classification of the analyzed sample as a neroli‐type citrus essential oil with a high content of linalool‐derived compounds, suggesting considerable potential for antimicrobial applications due to the well‐documented bioactive properties of oxygenated monoterpenes.

**TABLE 1:** Chemical composition of DFEO (percentage mean values ± SD).

No.	Compound	LRI ^calc^	LRI ^lit^	%
1	α‐Pinene	938	942	0.4 ± 0.02
2	Sabinene	965	966	0.2 ± 0.02
3	β‐Pinene	972	970	6.8 ± 0.15
4	Limonene	1028	1031	19.9 ± 1.11
5	*Cis*‐β‐ocimene	1037	1043	0.5 ± 0.03
6	*Cis*‐linalool oxide	1068	1070	0.3 ± 0.02
7	*Trans*‐linalool oxide	1085	1082	0.5 ± 0.03
8	Linalool	1100	1102	42.8 ± 3.12
9	Terpinen‐4‐ol	1178	1182	0.1 ± 0.02
10	α‐Terpineol	1195	1193	6.7 ± 0.12
11	Linalyl acetate	1250	1252	15.3 ± 1.06
12	*Cis*‐geraniol	1255	1254	1.1 ± 0.06
13	Nerol acetate	1338	1342	1.1 ± 0.04
14	Geranyl acetate	1362	1366	1.9 ± 0.05
15	β‐Elemene	1390	1387	0.1 ± 0.02
16	β‐Caryophyllene	1428	1431	1.3 ± 0.05
17	Humulene	1475	1473	0.1 ± 0.02
18	*Trans*‐nerolidol	1568	1567	0.6 ± 0.03
19	Spathulenol	1575	1576	tr
20	Caryophyllene oxide	1585	1580	0.1 ± 0.02
21	β‐Eudesmene	1655	1654	0.1 ± 0.02
	**Total**			99.9
	**Monoterpenes**			27.8
	**Oxygenated monoterpenes**			51.5
	**Sesquiterpenes**			2.2
	**Oxygenated sesquiterpenes**			0.1
	**Monoterpenoids**			18.3

*Note*: The components are reported according to their elution order on apolar column.

Abbreviations: LRI ^calc^, linear retention indices measured on apolar column; LRI ^lit^, linear retention indices from literature; tr, percentage mean values ≤ 0.1%.

### 3.2 Antimicrobial Activity of DFEO

#### 3.2.1 Disc Diffusion Assay

DFEO exhibited broad‐spectrum antimicrobial activity against all tested microorganisms, with inhibition zones ranging from 7.33 to 13.67 mm (Table 2). Gram‐positive bacteria showed the highest susceptibility, with inhibition zones ranging from 12.67 to 13.67 mm, followed by Gram‐negative bacteria (9.33–11.00 mm) and yeasts (7.33–8.67 mm). *E. faecalis* showed the highest susceptibility, with an average inhibition zone of 13.67 mm, followed by *S. aureus* (13.33 mm) and *L. monocytogenes* (12.67 mm). Among Gram‐negative bacteria, *E. coli* was the most susceptible species (11.00 mm), whereas *S. enterica* and *Y. enterocolitica* showed comparable susceptibility (9.33 and 9.67 mm, respectively).

**TABLE 2:** Antimicrobial activity (expressed as inhibition zone in mm) of antibiotics (ATB) and daidai flower essential oil (DFEO), evaluated by disc diffusion method.

Microorganism	DFEO	ATB
*Escherichia coli* CCM 3954	11.00 ± 1.00^bc,B^	30.33 ± 0.58^ab,A^
*Salmonella enterica* CCM 3807	9.33 ± 0.58^cde,B^	32.67 ± 0.58^a,A^
*Yersinia enterocolitica* CCM 7204^T^	9.67 ± 0.58 ^cd,B^	30.67 ± 0.58^b,A^
*Enterococcus faecalis* CCM 4224	13.67 ± 0.58^a,B^	31.67 ± 0.58^a,A^
*Listeria monocytogenes* CCM 4699	12.67 ± 0.58^a,B^	30.33 ± 0.58^ab,A^
*Staphylococcus aureus* CCM 4423	13.33 ± 0.58^a,B^	31.67 ± 0.58^a,A^
*Candida albicans* CCM 8186	7.33 ± 0.58^f,B^	29.33 ± 0.58^b,A^
*Candida glabrata* CCM 8270	7.67 ± 0.58^ef,B^	28.67 ± 0.58^c,A^
*Candida krusei* CCM 8271	8.67 ± 0.58^def,B^	29.67 ± 0.58^b,A^
*Candida parapsilosis* CCM 8260	8.33 ± 0.58^def,B^	30.67 ± 0.58^b,A^
*Candida tropicalis* CCM 8223	8.33 ± 0.58^def,B^	28.33 ± 0.58^c,A^

*Note*: Data are the mean (± standard deviation) of three replicates. Different superscript lowercase letters indicate statistically significant differences within each column (*p* ≤ 0.05). Different superscript uppercase letters indicate statistically significant differences within each row (*p* ≤ 0.05).

Abbreviations: ATB, antibiotics; DFEO, daidai flower essential oil.

Yeasts of the genus *Candida* demonstrated the lowest susceptibility, with inhibition zones ranging from 7.33 to 8.67 mm. The most resistant species was *C. albicans* (7.33 mm), whereas *C. krusei* exhibited the highest susceptibility within this group (8.67 mm).

In comparison with conventional antimicrobial agents used as positive controls (inhibition zones 28.33–32.67 mm), the antimicrobial activity of DFEO corresponded to approximately 25%–45% of the efficacy of the reference compounds, which is considered acceptable for a natural product.

#### 3.2.2 Minimum Inhibitory Concentration

The broth microdilution assay revealed pronounced differences in susceptibility among the tested microorganisms, with MIC_50_ values ranging from 0.29 to 6.30 mg/mL and MIC_90_ values from 1.43 to 8.52 mg/mL (Table 3).

**TABLE 3:** Minimal inhibition concentration (MIC_50_ and MIC_90_) of DFEO expressed in mg/mL.

Microorganism	MIC_50_	MIC_90_
*Escherichia coli* CCM 3954	1.57 ± 0.13^g^	3.87 ± 0.51^ef^
*Salmonella enterica* CCM 3807	2.69 ± 0.09^de^	4.65 ± 0.06^d^
*Yersinia enterocolitica* CCM 7204^T^	3.78 ± 0.18^b^	5.88 ± 0.11^c^
*Enterococcus faecalis* CCM 4224	6.30 ± 0.47^a^	8.52 ± 0.22^a^
*Listeria monocytogenes* CCM 4699	5.70 ± 0.28^a^	7.82 ± 0.15^a^
*Staphylococcus aureus* CCM 4423	2.57 ± 0.16^de^	4.77 ± 0.19^d^
*Candida albicans* CCM 8186	0.29 ± 0.05^h^	1.43 ± 0.29^g^
*Candida glabrata* CCM 8270	1.67 ± 0.27^fg^	3.54 ± 0.26^f^
*Candida krusei* CCM 8271	3.54 ± 0.06^bc^	5.70 ± 0.31^c^
*Candida parapsilosis* CCM 8260	3.03 ± 0.08^cd^	7.00 ± 0.12^b^
*Candida tropicalis* CCM 8223	2.20 ± 0.16^ef^	4.55 ± 0.14^de^

*Note*: Data are the mean (± standard deviation) of three replicates. Different superscript lowercase letters indicate statistically different values within the column (*p* ≤ 0.05).

The highest susceptibility was observed for *C. albicans*, which exhibited the lowest MIC_50_ (0.29 mg/mL) and MIC_90_ (1.43 mg/mL) values. The apparent discrepancy between the disc diffusion assay and MIC determination may be explained by the limited diffusion of hydrophobic essential oil components in agar media, which can underestimate antimicrobial activity in diffusion‐based assays. *E. coli* also demonstrated relatively high susceptibility, with MIC_50_ and MIC_90_ values of 1.57 mg/mL and 3.87 mg/mL, respectively.

Gram‐positive bacteria exhibited variable susceptibility. *S. aureus* showed relatively high sensitivity (MIC_50_ = 2.57 mg/mL), whereas *E. faecalis* and *L. monocytogenes* displayed the highest MIC_50_ values (6.30 and 5.70 mg/mL, respectively).

Among the yeast species, susceptibility varied considerably. *C. tropicalis* (MIC_50_ = 2.20 mg/mL) and *C. glabrata* (MIC_50_ = 1.67 mg/mL) demonstrated moderate susceptibility, whereas *C. krusei* (MIC_50_ = 3.54 mg/mL) and *C. parapsilosis* (MIC_50_ = 3.03 mg/mL) were less susceptible to DFEO.

### 3.3 Antimicrobial Activity in the Vapor Phase

#### 3.3.1 Pear Model

**FIGURE 2:**
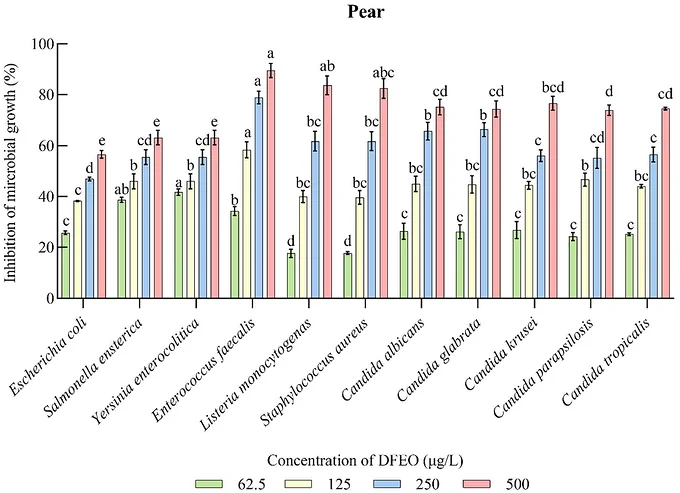
In situ analysis of the inhibition of microbial growth (%) by the vapor phase of DFEO at different concentrations in pear. Data are the mean (bars indicate ± standard deviation) of three samples. Different lowercase letters (a–e) indicate statistically significant differences within each EO concentration (*p* ≤ 0.05).

Antimicrobial activity of DFEO was evaluated using an in situ vapor‐phase method on a pear model against selected bacterial and yeast strains at concentrations of 500, 250, 125, and 62.5 µg/L. The obtained results indicate a strong concentration‐dependent inhibitory effect, with decreasing concentrations resulting in a gradual reduction of microbial growth inhibition (Figure 2).

At the highest tested concentration (500 µg/L), inhibition values ranged from approximately 56.63% to 89.47%. The most susceptible microorganism was *E. faecalis*, showing 89.47% inhibition. High susceptibility was also observed for *L. monocytogenes* (83.67%) and *S. aureus* (82.44%). In contrast, Gram‐negative bacteria exhibited lower susceptibility, particularly *E. coli* (56.63%) and *S. enterica* (63.17%).

At a concentration of 250 µg/L, inhibition ranged between 46.92% and 78.90%. The highest inhibition was again observed for *E. faecalis* (78.90%), while the lowest value was recorded for *E. coli* (46.92%). A similar trend was observed at a concentration of 125 µg/L, where inhibition values ranged from 38.27% to 58.41%. At the lowest tested concentration (62.5 µg/L), inhibition ranged from 17.58% to 34.29%, with *E. faecalis* again showing the highest susceptibility.

Yeasts of the genus *Candida* showed moderate susceptibility to DFEO. At a concentration of 500 µg/L, inhibition ranged from approximately 73.83% to 76.63%, with the highest inhibition observed for *C. krusei* (76.63%). As the concentration decreased, a gradual reduction in inhibitory activity was also observed for yeast strains.

Overall, DFEO exhibited stronger inhibitory activity against Gram‐positive bacteria than Gram‐negative bacteria in the pear model, while *Candida* species showed moderate susceptibility. These results confirm the concentration‐dependent antimicrobial activity of DFEO.

#### 3.3.2 Peach Model

**FIGURE 3:**
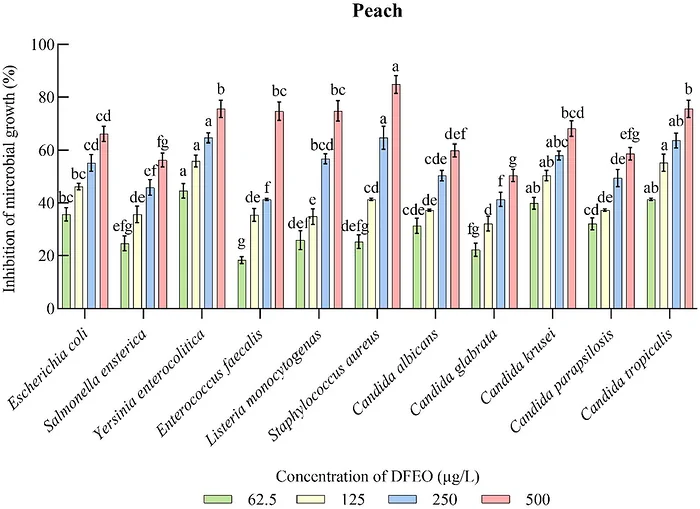
In situ analysis of the inhibition of microbial growth (%) by the vapor phase of DFEO at different concentrations in peach. Data are the mean (bars indicate ± standard deviation) of three samples. Different lowercase letters (a–f) indicate statistically significant differences within each EO concentration (*p* ≤ 0.05).

Antimicrobial activity of DFEO was also evaluated using an in situ method on a peach model at concentrations of 500, 250, 125, and 62.5 µg/L against selected bacterial and yeast strains. The results indicate that the efficacy of DFEO was strongly influenced by concentration, with decreasing concentrations leading to a gradual reduction in microbial inhibition (Figure 3).

At a concentration of 500 µg/L, inhibition ranged between approximately 50.47% and 84.83%. The highest inhibition was recorded for *S. aureus* (84.83%). High susceptibility was also observed for *Y. enterocolitica* (75.59%), *C. tropicalis* (75.59%), *L. monocytogenes* (74.81%), and *E. faecalis* (74.72%). Lower inhibition values were observed mainly for *C. glabrata* (50.47%).

At a concentration of 250 µg/L, inhibition ranged between 41.33% and 64.65%. The highest inhibitory activity was again recorded for *S. aureus* (64.65%), while the lowest value was observed for *E. faecalis* (41.33%). At a concentration of 125 µg/L, inhibition ranged between approximately 29.27% and 58.33%, with higher values mainly observed for *Y. enterocolitica* and *C. tropicalis*.

At the lowest concentration (62.5 µg/L), inhibition ranged from approximately 18.68% to 44.64%. The highest susceptibility at this concentration was observed for *Y. enterocolitica*, whereas the lowest inhibition values were recorded for *E. faecalis*.

Yeasts of the genus *Candida* showed moderate susceptibility to DFEO, with inhibition values ranging from 50.47% to 75.59% at the highest concentration, where the strongest inhibition was observed for *C. tropicalis*.

#### 3.3.3 Kohlrabi Model

**FIGURE 4:**
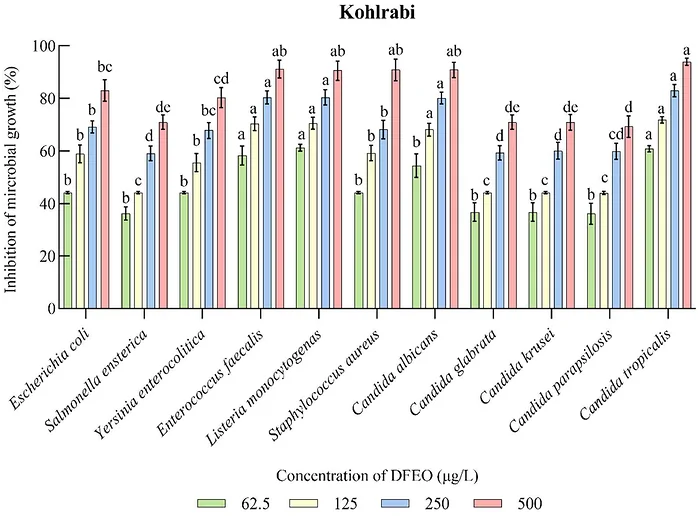
In situ analysis of the inhibition of microbial growth (%) by the vapor phase of DFEO at different concentrations in kohlrabi. Data are the mean (bars indicate ± standard deviation) of three samples. Different lowercase letters (a–e) indicate statistically significant differences within each EO concentration (*p* ≤ 0.05).

Antimicrobial activity of DFEO was further evaluated using an in situ method on a kohlrabi model at concentrations of 500, 250, 125, and 62.5 µg/L against selected bacterial and yeast strains. The results confirmed a clear concentration‐dependent effect, with decreasing concentrations resulting in a gradual reduction of antimicrobial activity (Figure 4).

At the highest concentration (500 µg/L), inhibition ranged between approximately 69.32% and 93.89%. The highest inhibition was recorded for *C. tropicalis* (93.89%), while high inhibition values were also observed for *E. faecalis* (91.21%), *S. aureus* (90.85%), *C. albicans* (90.87%), and *L. monocytogenes* (90.59%). Lower inhibition values were observed for *C. parapsilosis* (69.32%) and *C. krusei* (70.88%).

At a concentration of 250 µg/L, inhibition ranged between 59.15% and 83.02%, with the highest susceptibility again observed for *C. tropicalis* (83.02%). At a concentration of 125 µg/L, inhibition values ranged between 43.98% and 71.82%, with the highest inhibition again observed for *C. tropicalis*.

At the lowest concentration (62.5 µg/L), inhibition ranged between 36.17% and 61.13%, with the highest value observed for *L. monocytogenes* (61.13%) and the lowest for *C. parapsilosis* (36.17%).

#### 3.3.4 Potato Model

**FIGURE 5:**
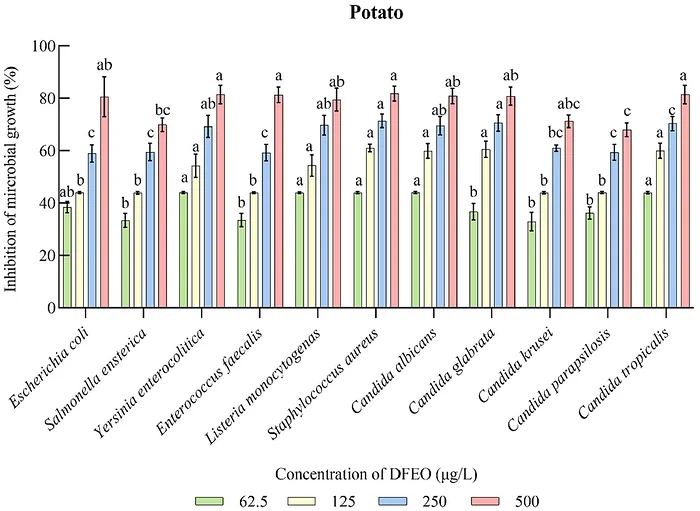
In situ analysis of the inhibition of microbial growth (%) by the vapor phase of DFEO at different concentrations in potato. Data are the mean (bars indicate ± standard deviation) of three samples. Different lowercase letters (a–c) indicate statistically significant differences within each EO concentration (*p* ≤ 0.05).

The antimicrobial activity of DFEO was also evaluated on a potato model under the same experimental conditions. The results confirmed a concentration‐dependent inhibitory effect against all tested microorganisms (Figure 5).

At a concentration of 500 µg/L, inhibition ranged between approximately 68.01% and 81.84%. The highest inhibition values were recorded for *S. aureus* (81.84%), *C. tropicalis* (81.48%), *E. faecalis* (81.41%), and *C. glabrata* (80.88%).

At a concentration of 250 µg/L, inhibition ranged between 58.97% and 71.39%, with the highest inhibition again observed for *S. aureus* (71.39%). At a concentration of 125 µg/L, inhibition ranged between 43.98% and 61.08%, with higher values observed for *S. aureus* and *C. glabrata*.

At the lowest concentration (62.5 µg/L), inhibition ranged between 29.07% and 44.15%, with the highest value observed for *Y. enterocolitica* (44.09%).

#### 3.3.5 Comparison of Food Models

When comparing the individual models, it can be concluded that the antimicrobial activity of DFEO was influenced not only by concentration but also by the type of food matrix. In general, the highest inhibition values were observed in the kohlrabi model, whereas lower inhibition levels were recorded in some cases in the peach model. The pear model showed pronounced susceptibility particularly for Gram‐positive bacteria, while in the potato model inhibition values were relatively balanced among the tested microorganisms. Across all models, a clear concentration‐dependent trend was observed, with decreasing DFEO concentrations resulting in progressively reduced antimicrobial activity.

**FIGURE 6:**
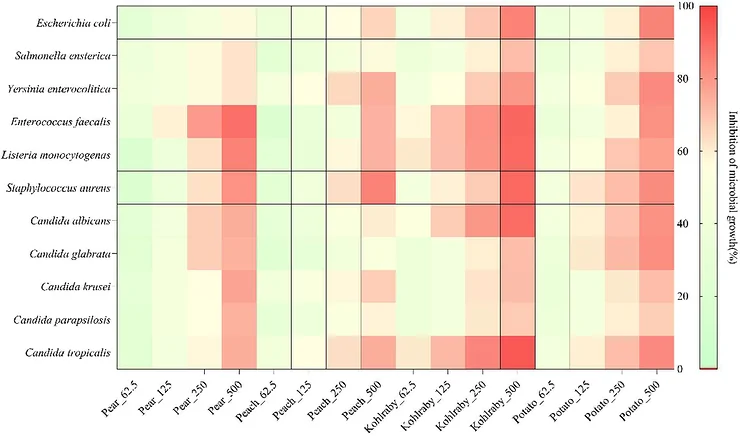
Heatmap illustrating the in situ antimicrobial activity (%) of daidai flower essential oil (DFEO) in pear, peach, kohlrabi, and potato model systems. The inhibition of microbial growth is shown for 12 microorganisms at four concentrations (62.5, 125, 250, and 500 µg/L). Labels on the *x*‐axis represent combinations of the tested food model and DFEO concentration.

These differences in antimicrobial efficacy among the tested food matrices are summarized in the heatmap shown in Figure 6. The visualization highlights the variability in microbial susceptibility across different models and concentrations of DFEO, providing an overview of the relative inhibitory effects observed in the in situ experiments.

### 3.4 Microbiological Quality of Arugula During Storage

While the antimicrobial activity of DFEO in the vapor phase was initially evaluated using several model plant matrices to simulate contaminated food surfaces, the preservation potential of the essential oil was subsequently assessed under real storage conditions using fresh arugula as the target food matrix.

The antimicrobial effect of DFEO was therefore further evaluated on an arugula model during storage for 14 days, with changes in coliform bacteria and TVC monitored (Table 4). The experiment included four treatment variants: control, control with vacuum packaging, EO application, and EO combined with vacuum packaging (Table 5). Since shelf life also depends on sensory quality, appearance, texture, odor, and acceptability, which were not assessed in the present study, the obtained results support improved microbiological quality during refrigerated storage rather than full shelf‐life extension.

**TABLE 4:** Results of two‐way ANOVA for the effects of treatment and storage time on coliform bacteria (TC) and total viable counts (TVC) in arugula during refrigerated storage.

Source of variation	df	TC		TVC	
		*F* value	*p* value	*F* value	*p* value
Treatment	3	44.696	< 0.001	172.029	< 0.001
Storage time	2	32.137	< 0.001	72.534	< 0.001
Treatment × storage time	6	1.644	0.179	0.853	0.543
Error	24				

*Note*: The significance level was set at *p* ≤ 0.05.

Abbreviation: df, degrees of freedom.

**TABLE 5:** Arugula experiment of coliform bacteria and total viable counts across treatment groups (control, control vacuum, control EO, EO vacuum) at 1, 7, and 14 days.

Group	1 day		7 days		14 days	
	TC	TVC	TC	TVC	TC	TVC
C1	2.52 ± 0.08^a^	3.59 ± 0.20^a^	2.78 ± 0.11^a^	3.86 ± 0.17^a^	3.49 ± 0.41^a^	4.32 ± 0.04^a^
C2	2.45 ± 0.17^a^	2.98 ± 0.11^b^	2.62 ± 0.20^a^	3.28 ± 0.15^b^	2.87 ± 0.09^ab^	3.47 ± 0.16^b^
C3	1.82 ± 0.07^b^	2.64 ± 0.09^bc^	2.36 ± 0.44^ab^	2.84 ± 0.08^c^	2.53 ± 0.21^cd^	3.18 ± 0.13^c^
EO	1.59 ± 0.06^b^	2.37 ± 0.13^c^	1.78 ± 0.08^b^	2.68 ± 0.06^c^	2.23 ± 0.13^d^	3.04 ± 0.06^c^

*Note*: Different superscript lowercase letters indicate statistically different values within the column for each microbial parameter separately (*p* ≤ 0.05). TC and TVC are independent microbiological endpoints; no statistical comparison was performed between the two parameters.

After 1 day of storage, the highest values of coliform bacteria were recorded in the control sample (2.52 log CFU/g), whereas the lowest values were observed in the EO combined with vacuum packaging treatment (1.59 log CFU/g). A similar trend was observed for total viable microorganisms, where the control sample reached 3.59 log CFU/g, while EO vacuum treatment showed the lowest microbial load (2.37 log CFU/g).

After 7 days of storage, microbial populations increased in all treatments. Coliform bacteria in the control sample reached 2.78 log CFU/g, while the lowest values were again observed in the EO vacuum treatment (1.78 log CFU/g). A similar trend was observed for total viable microorganisms, where the control reached 3.86 log CFU/g, whereas the combination of EO and vacuum packaging showed lower values (2.68 log CFU/g).

After 14 days of storage, a further increase in microbial populations was observed across all treatments. The highest coliform counts were recorded in the control sample (3.49 log CFU/g), while the lowest values were detected in the EO vacuum treatment (2.23 log CFU/g). A similar pattern was observed for total viable microorganisms, where the control reached 4.32 log CFU/g, while EO vacuum treatment showed the lowest values (3.04 log CFU/g) Table 5.

**FIGURE 7:**
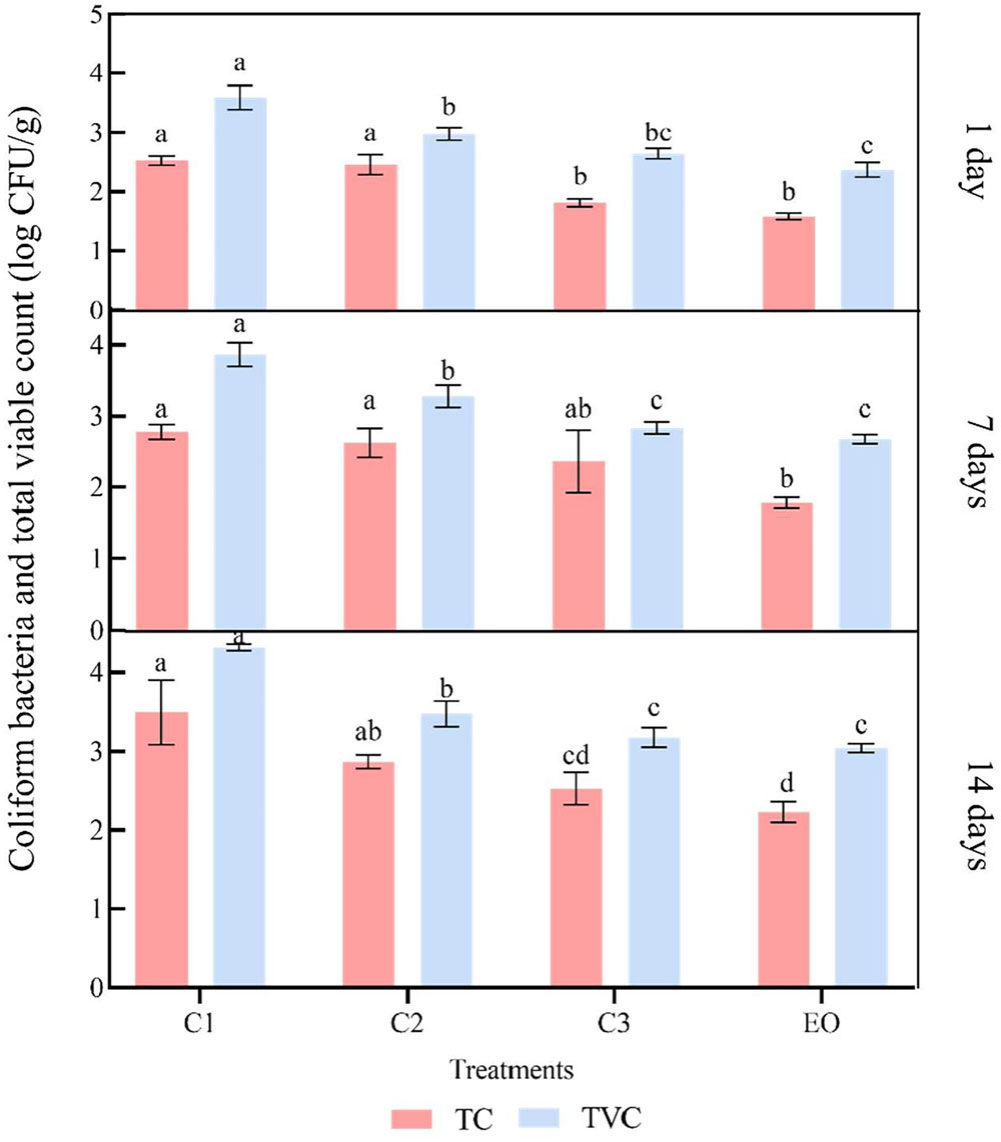
Arugula experiment of coliform bacteria and total viable counts across treatment groups (control, control vacuum, control EO, EO vacuum) at 1, 7, and 14 days. Data are the mean (bars indicate ± standard deviation) of three samples. Different superscript lowercase letters indicate statistically different values within the column (*p* ≤ 0.05). Letters are not comparable between TC and TVC, as statistical comparisons were performed separately for each microbial parameter. Abbreviations: TC, coliform bacteria count; TVC, total viable count.

The statistical significance of the main effects and their interaction was evaluated by two‐way analysis of variance (Table 4). For both TC and TVC, the main effects of treatment and storage time were statistically significant (*p* < 0.001), indicating that both the preservation strategy and storage duration strongly influenced the microbial load of arugula. However, the treatment × storage time interaction was not significant for either parameter (TC: *p* = 0.179; TVC: *p* = 0.543), meaning that the relative antimicrobial performance of the four treatments remained consistent across the entire 14 days refrigerated storage period. The obtained results indicate that the application of DFEO in combination with vacuum packaging exerted the most pronounced inhibitory effect on microbial growth during arugula storage. This treatment consistently resulted in the lowest values of both coliform bacteria and total viable microorganisms throughout the storage period. In contrast, the highest microbial populations were observed in untreated control samples (Figure 7). The observed inhibitory effect can likely be attributed to the combined action of DFEO and vacuum packaging. While EO application alone reduced microbial populations compared with the control, the most pronounced effect was observed when EO was combined with vacuum packaging. The vacuum environment likely limits oxygen availability and thereby slows the growth of aerobic microorganisms, while bioactive compounds present in the essential oil may disrupt microbial cell membranes and interfere with metabolic processes. The inhibitory advantage of the EO‐treated vacuum package remained consistent throughout the 14‐day refrigerated storage period, with consistently lower counts of both coliform bacteria and total viable microorganisms compared with the other three treatment groups (Table 5).

**FIGURE 8:**
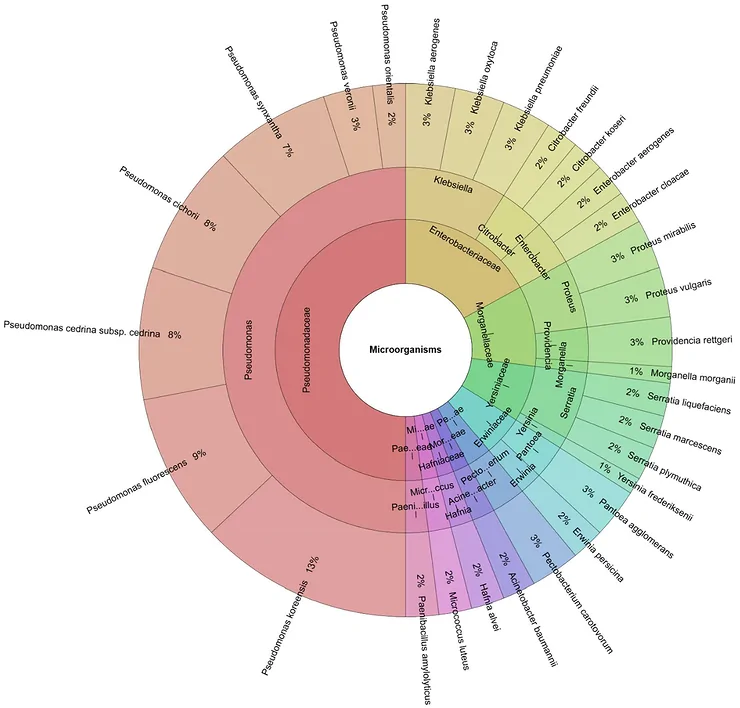
Distribution of bacterial species among selected culturable isolates identified from arugula by MALDI‐TOF MS. As colonies were selected morphologically without a standardized per‐treatment/time sampling protocol, the reported values reflect the composition of the selected isolate set rather than the true relative abundance of species in the arugula microbiota.

In total, 263 bacterial isolates were identified, belonging to 9 families, 14 genera, and 30 species among the selected culturable isolates, with the distribution shown in Figure 8, comprising 123 isolates from the control group, 44 from the control with vacuum packaging, 82 from the DFEO‐treated group, and 14 from the DFEO with vacuum packaging group, obtained at Day 1 (*n* = 35), Day 7 (*n* = 89), and Day 14 (*n* = 139) of storage. The highest representation was observed for the family Pseudomonadaceae, which accounted for 49.4% of the selected culturable isolates (130 isolates). The second most abundant group comprised members of the family Enterobacteriaceae (16.4%; 43 isolates), followed by Morganellaceae (9.9%; 26 isolates) and Yersiniaceae (7.2%; 19 isolates). Less abundant families included Erwiniaceae (4.9%; 13 isolates), Pectobacteriaceae (3.4%; 9 isolates), Hafniaceae, Micrococcaceae, and Paenibacillaceae (each approximately 2.3%; 6 isolates), and Moraxellaceae (1.9%; 5 isolates).

At the genus level, *Pseudomonas* was dominant, comprising seven identified species. The most frequently detected species was *Pseudomonas koreensis* (34 isolates; 12.9%), followed by *P. fluorescens* (24 isolates; 9.1%), *Pseudomonas cedrina* (22 isolates; 8.4%), and *Pseudomonas cichorii* (20 isolates; 7.6%). Other species of this genus included *Pseudomonas synxantha* (18 isolates; 6.8%), *Pseudomonas veronii* (7 isolates; 2.7%), and *Pseudomonas orientalis* (5 isolates; 1.9%).

Among the Enterobacteriaceae, the identified species included *Klebsiella aerogenes* (eight isolates; 3.0%), *Klebsiella pneumoniae* (eight isolates; 3.0%), *Klebsiella oxytoca* (seven isolates; 2.7%), *Enterobacter aerogenes* (six isolates; 2.3%), and *Enterobacter cloacae* (five isolates; 1.9%). Additional species included *Citrobacter freundii* (five isolates; 1.9%) and *Citrobacter koseri* (four isolates; 1.5%).

To a lesser extent, species belonging to other families were also detected, including *Proteus mirabilis* (seven isolates; 2.7%), *Proteus vulgaris* (eight isolates; 3.0%), *Providencia rettgeri* (eight isolates; 3.0%), *Serratia plymuthica* (six isolates; 2.3%), *Serratia liquefaciens* (five isolates; 1.9%), and *Serratia marcescens* (five isolates; 1.9%). Additional species identified included *Pantoea agglomerans* (seven isolates; 2.7%), *Pectobacterium carotovorum* (nine isolates; 3.4%), *Hafnia alvei* (six isolates; 2.3%), *Paenibacillus amylolyticus* (six isolates; 2.3%), *Micrococcus luteus* (six isolates; 2.3%), *Acinetobacter baumannii* (five isolates; 1.9%), and *Yersinia frederiksenii* (three isolates; 1.1%).

These results indicate a predominance of Gram‐negative psychrotrophic bacteria, particularly species of the genus *Pseudomonas*, which represent a typical component of the cultivable bacterial isolate profile of leafy vegetables and play an important role in the spoilage of fresh plant products during storage.

The occurrence of individual bacterial species isolated from arugula under different treatment conditions is presented in Table 6. Most identified species were detected in both control and vacuum‐packaged samples, indicating a relatively high diversity of the natural microbiota of arugula. The application of EO, particularly in combination with vacuum packaging, resulted in a reduction in the occurrence of several bacterial species. While some species, such as *Acinetobacter baumannii*, *Citrobacter freundii*, *Citrobacter koseri*, and *Enterobacter cloacae*, were detected across several treatment variants, a decrease in the occurrence of several *Pseudomonas* species and some members of the family Enterobacteriaceae was observed in EO‐treated samples. These findings suggest that EO application, especially when combined with vacuum packaging, may contribute to the suppression of part of the natural microbiota associated with arugula.

**TABLE 6:** Occurrence of bacterial species isolated from arugula under various treatment conditions (control, vacuum packaging, application of essential oil, and a combination of essential oil and vacuum packaging).

Microorganisms	Control	Control + vacuum	Control + EO	EO + vacuum
*Acinetobacter baumannii*	1	1	1	2
*Citrobacter freundii*	1	2	1	1
*Citrobacter koseri*	1	1	1	1
*Enterobacter aerogenes*	2	3	1	0
*Enterobacter cloacae*	1	2	1	1
*Erwinia persicina*	2	2	1	1
*Hafnia alvei*	1	1	3	1
*Klebsiella aerogenes*	2	3	1	2
*Klebsiella oxytoca*	2	2	3	0
*Klebsiella pneumoniae*	2	2	2	2
*Micrococcus luteus*	2	2	2	0
*Morganella morganii*	1	1	1	0
*Paenibacillus amylolyticus*	2	3	1	0
*Pantoea agglomerans*	3	3	1	0
*Pectobacterium carotovorum*	3	4	2	0
*Proteus mirabilis*	2	2	3	0
*Proteus vulgaris*	4	2	2	0
*Providencia rettgeri*	3	2	3	0
*Pseudomonas cedrina* subsp. *cedrina*	12	0	10	0
*Pseudomonas cichorii*	11	0	9	0
*Pseudomonas fluorescens*	18	0	6	0
*Pseudomonas koreensis*	22	0	12	0
*Pseudomonas orientalis*	3	0	2	0
*Pseudomonas synxantha*	7	6	5	0
*Pseudomonas veronii*	3	0	2	2
*Serratia liquefaciens*	3	0	1	1
*Serratia marcescens*	3	0	2	0
*Serratia plymuthica*	4	0	2	0
*Yersinia frederiksenii*	2	0	1	0

### 3.5 Insecticidal Activity

DFEO showed a pronounced concentration‐dependent insecticidal activity against both tested bruchid species, with comparable efficacy between *C. maculatus* and *M. dorsalis* (Table 7). At the highest tested concentration (100%), daidai EO exhibited strong insecticidal effects with mortality rates of 95.33% for *C. maculatus* and 93.67% for *M. dorsalis*. Mortality decreased gradually with decreasing concentrations. At 50% concentration, mortality reached 76.00% and 79.00%, respectively, while at 25% mortality decreased to 57.00% and 50.67%. At the lowest tested concentration (3.125%), mortality declined to 14.00% and 6.33%.

**TABLE 7:** Mortality (%) at different concentrations of DFEO.

Concentration of DFEO (%)	*C. maculatus*	*M. dorsalis*
3.125	14.00 ± 5.29^e^	6.33 ± 1.15^f^
6.25	21.67 ± 3.51^de^	18.67 ± 2.08^e^
12.5	31.00 ± 2.00^d^	28.00 ± 5.57^d^
25	57.00 ± 2.65^c^	50.67 ± 3.06^c^
50	76.00 ± 3.46^b^	79.00 ± 2.65^b^
100	95.33 ± 3.21^a^	93.67 ± 1.15^a^

*Note*: Data represent the mean (± standard deviation) of three replicates. Different superscript lowercase letters indicate statistically different values within the column (*p* ≤ 0.05).

The calculated lethal concentrations confirmed the high susceptibility of both species to daidai EO. The LC_50_ values were estimated at 18.09% for *C. maculatus* and 20.85% for *M. dorsalis* (95% CI: 16.57–19.74 and 19.24–22.58, respectively), while LC_90_ values reached 98.80% and 92.57% (95% CI: 83.59–116.77 and 79.95–107.18, respectively) (Table 8).

**TABLE 8:** LC_50_ and LC_90_ values of daidai flower essential oil (DFEO) against *Callosobruchus maculatus* and *Megabruchidius dorsalis*.

	LC_50_ (%)	95% CI	LC_90_ (%)	95% CI
*C. maculatus*	18.09	16.57–19.74	98.80	83.59.76–116.77
*M. dorsalis*	20.85	19.24–22.58	92.57	79.95–107.18

*Note*: The upper bound of the 95% CI for LC_90_ in *C. maculatus* exceeds 100% due to the LC_90_ estimate lying close to the highest tested concentration (undiluted oil); this reflects statistical extrapolation uncertainty inherent to probit regression rather than a physically achievable concentration.

## 4 Discussion

### 4.1 Chemical Characterization and Chemotype Variability

Chemical analysis of the DFEO revealed a profile that differs from many commercially available citrus essential oils, highlighting the chemotypic diversity within the *C. aurantium* complex. The dominance of linalool (42.8%), linalyl acetate (15.3%), and limonene (19.9%) classifies this essential oil as a neroli‐type chemotype. This observation is consistent with phylogeographic studies by Liang et al. (2021) and González‐Mas et al. (2019), who reported similar chemical profiles in *C. aurantium* var. *daidai* cultivated in different climatic regions of China.

It should be noted that the GC–MS analysis performed in this study is semiquantitative, as relative peak‐area percentages were calculated without the use of an internal standard or correction factors. The reported percentages should therefore be interpreted with caution, and direct quantitative links between exact compound percentages and biological activity require further validation.

Furthermore, only one commercial batch of DFEO (batch no. 20240512), chemically characterized by GC–MS analysis in this study, was evaluated. Given that essential oil composition is known to vary considerably depending on geographical origin, harvest season, and extraction method, the antimicrobial and insecticidal findings reported here should be interpreted as reflecting the properties of this specific, chemically characterized batch, and confirmation of their broader applicability across different DFEO batches would require testing of additional independently sourced batches.

Compared with other citrus essential oils, the neroli chemotype differs substantially from bergamot‐type oils, which are typically dominated by linalyl acetate, or sweet orange oils characterized by high limonene content. These differences suggest cultivar‐specific biosynthetic pathways influencing monoterpene production in *C. aurantium* (Benelli et al. 2018; García‐Salinas et al. 2018).

The high proportion of oxygenated compounds (69.8%, comprising oxygenated monoterpenes 51.5% and monoterpenoids 18.3%) relative to monoterpene hydrocarbons (27.3%) and sesquiterpenes (2.1%) may explain the pronounced antimicrobial activity observed in this study. Oxygenated monoterpenes are widely recognized as the most biologically active components of essential oils and have consistently demonstrated stronger antimicrobial effects than hydrocarbon monoterpenes (García‐Salinas et al. 2018; Guimarães et al. 2019). Comparable proportions of oxygenated monoterpenes have been reported in neroli‐type essential oils from *C. aurantium* var. *amara* grown in Tunisia and Morocco, suggesting a relatively conserved chemical profile of neroli‐type oils across different geographical regions (Ellouze et al. 2012).

The relatively low abundance of sesquiterpenes (β‐caryophyllene 1.3%, *trans*‐nerolidol 0.6%, and caryophyllene oxide 0.1%) suggests that the antimicrobial activity of DFEO is primarily associated with monoterpene constituents. Fractionation studies have demonstrated that monoterpene‐rich fractions often exhibit stronger antimicrobial effects than sesquiterpene fractions of the same essential oil (Guimarães et al. 2019). Nevertheless, minor components such as *trans*‐nerolidol may still contribute to overall biological activity through synergistic interactions with major compounds such as linalool (Basavegowda and Baek 2021).

The stability of the chemical profile is an important factor for potential practical applications of essential oils. Monoterpene alcohols such as linalool are susceptible to oxidation and structural transformation under conditions of light exposure and elevated temperatures, which may lead to reduced biological activity or altered sensory properties (An et al. 2021). No stability experiment was conducted in the present study. However, An et al. (2021) reported that the chemical profile of linalool‐rich essential oils remained stable for 6 months under refrigerated storage conditions (4°C, dark storage), suggesting that the chemical composition of DFEO as used in this study was unlikely to have changed significantly during the experimental period. It should be noted, however, that only a single commercial batch of DFEO (batch no. 20240512) was used in this study. Since essential oil composition can vary depending on plant origin, harvest season, extraction method, and storage conditions, the biological activities reported here reflect the properties of this specific batch and may not be fully representative of DFEO in general. In addition, a Tween 80‐only control group was not included in the arugula storage experiment; therefore, matrix‐specific effects of the emulsifier on leaf surface wetting or microbial recovery cannot be fully excluded based solely on literature data from other systems.

Furthermore, the three replicates within each treatment group were subsamples obtained from a single commercial arugula lot rather than independent biological replicates from separate lots; the experimental unit therefore corresponds to a technical replicate, and confirmation across independently sourced arugula lots would be required to generalize these findings beyond this specific lot.

Additionally, colony selection for MALDI‐TOF MS identification was based on morphological criteria without a fixed number of isolates per treatment group or sampling time point; consequently, the unequal isolate numbers obtained across groups may partly reflect differences in sampling effort rather than true differences in microbial abundance, and the reported data should be interpreted as the distribution of selected culturable isolates rather than a quantitative measure of relative abundance.

### 4.2 Antimicrobial Mechanisms and Structure–Activity Relationships

Antimicrobial testing revealed clear differences in susceptibility between Gram‐positive and Gram‐negative bacteria, reflecting fundamental differences in cell wall architecture and resistance mechanisms. The MIC_50_ values obtained for Gram‐positive bacteria (*S. aureus*, *L. monocytogenes*, and *E. faecalis*) were comparable to those reported by Long et al. (2025) for purified linalool against clinical bacterial isolates. Similar MIC ranges have also been documented for linalool‐rich essential oils derived from other plant species, such as *Lavandula angustifolia* (Mijatovic et al. 2022), supporting the consistent antimicrobial activity of this compound across different botanical sources.

The higher resistance observed in Gram‐negative bacteria is commonly attributed to the presence of an outer membrane rich in lipopolysaccharides, which acts as a permeability barrier against hydrophobic compounds such as monoterpenes. Similar patterns of reduced susceptibility of Gram‐negative bacteria have been reported in studies investigating monoterpene alcohols and essential oils (Guimarães et al. 2019; Zengin and Baysal 2014). Interestingly, *E. coli* showed relatively high susceptibility compared with other Gram‐negative bacteria, which may reflect strain‐specific differences in membrane composition or efflux pump activity, as previously suggested by Guo et al. (2021).

From a food safety perspective, the relatively higher resistance of Gram‐negative bacteria is particularly relevant, as the majority of important foodborne pathogens, including *S. enterica, E. coli*, and *Y. enterocolitica*, belong to this group. Nevertheless, the MIC_50_ values obtained for *S. enterica* (2.69 mg/mL) and *E. coli* (1.57 mg/mL) in the present study indicate that DFEO retains meaningful antimicrobial activity against these pathogens, suggesting that higher application concentrations or synergistic combinations with other preservation strategies may be necessary to achieve effective control of Gram‐negative foodborne pathogens in practical food applications.

Structure–activity relationships among monoterpene alcohols indicate that the presence and position of the hydroxyl group play a key role in antimicrobial activity. Linalool, which contains a tertiary alcohol functional group, often demonstrates stronger antimicrobial effects than primary alcohols such as geraniol or citronellol (Guimarães et al. 2019). It has been suggested in the literature that ester derivatives such as linalyl acetate may contribute to prolonged antimicrobial activity through gradual hydrolysis and release of the active alcohol (Mączka et al. 2022); however, this was not directly tested in the present study.

Yeast isolates showed heterogeneous susceptibility, with the highest sensitivity observed in *C. albicans*. The MIC_50_ value obtained in this study (0.29 mg/mL) is consistent with previous reports describing strong antifungal activity of linalool‐rich essential oils against *C. albicans* (Medeiros et al. 2022). Similar trends were reported by Biernasiuk and Malm (2023), who observed higher susceptibility of *C. albicans* compared with non‐albicans *Candida* species.

The higher resistance of *C. krusei* and *C. glabrata* may reflect their intrinsic resistance mechanisms to azole antifungal agents, which may also confer partial tolerance to terpene‐based compounds. Karpiński et al. (2023) reported comparable MIC ranges for these species when exposed to essential oils rich in oxygenated monoterpenes. In addition, Fernandes et al. (2022) suggested overexpression of efflux transporters as a possible mechanism contributing to reduced susceptibility in these yeasts.

### 4.3 In Situ Antimicrobial Activity and Food Model Applications

The in situ experiments conducted on the pear model provided important insights into the practical antimicrobial potential of DFEO under conditions that more closely simulate real food systems. The concentration‐dependent antimicrobial activity observed in this study is consistent with results reported by Zhan et al. (2022), who demonstrated significant inhibition of *E. faecalis* in a raw chicken breast food model following application of essential oil at comparable concentrations.

Microbial responses in in situ conditions reflect not only intrinsic susceptibility to antimicrobial compounds but also the influence of the food matrix. Interactions between essential oil components and food constituents such as proteins, lipids, or carbohydrates may reduce the availability of active compounds and thus affect antimicrobial efficacy (Gómez‐Llorente et al. 2024; Zheng et al. 2024). Despite these interactions, the present results confirm that DFEO retains significant antimicrobial activity in complex food matrices.

The influence of pH is another important factor affecting essential oil activity. Acidic conditions typical for fruit matrices may enhance the antimicrobial effect of certain compounds by destabilizing microbial membranes (Noui Mehidi et al. 2024). The pear matrix used in this study, with a typical pH range of approximately 4.0–4.2, therefore provides favorable conditions for antimicrobial activity of monoterpenes. Similar pH‐dependent effects have been documented by Noui Mehidi et al. (2024), who reported increased antimicrobial activity of linalool and other terpene compounds at pH 4.0 compared to pH 7.0. In addition, the high water activity typical for fresh fruit facilitates diffusion of hydrophobic compounds to microbial cells (Zheng et al. 2024).

### 4.4 Microbiota of Arugula and Storage Applications

The storage experiment conducted on fresh arugula provided insights into the application of DFEO in postharvest preservation of leafy vegetables. It should be acknowledged that the microbiological analysis was limited to TVCs and coliform bacteria. Additional microbial groups such as psychrotrophic bacteria, yeasts and molds, lactic acid bacteria, or Enterobacteriaceae would provide a more comprehensive assessment of microbiological quality, as recommended for fresh leafy vegetables (Sequino et al. 2022). The current results should therefore not be interpreted as broad food safety evidence, but rather as preliminary data indicating the potential of DFEO for improving microbiological quality during refrigerated storage. Although several studies have characterized the microbiota of leafy greens using 16S rRNA sequencing, information about the culturable microbiota of arugula during storage remains limited. The combination of cultivation‐based methods with MALDI‐TOF MS identification therefore allows more precise identification of bacterial species associated with arugula during storage and evaluation of their response to natural antimicrobial treatments.

The MALDI‐TOF MS analysis of culture‐dependent bacterial isolates revealed a dominance of species of the genus *Pseudomonas*, particularly *P. koreensis* and P*. fluorescens*. Similar microbial profiles have been reported in metagenomic studies of leafy vegetable phyllosphere microbiota (Berg et al. 2020; Wassermann et al. 2019). Sequino et al. (2022) also documented a high prevalence of these species in arugula samples obtained from commercial retail sources.

The presence of *P. fluorescens* is particularly relevant from a food quality perspective, as this species is known to produce proteolytic and lipolytic enzymes responsible for spoilage and development of off‐flavor compounds during storage. Kumar et al. (2019) identified *P. fluorescens* as a major contributor to spoilage processes in refrigerated leafy vegetables, while Ding et al. (2025) demonstrated the ability of this species to produce volatile compounds such as indole and skatole under refrigerated conditions.

The detection of *Pantoea agglomerans* is also noteworthy, as this species has been reported as a potential reservoir of antibiotic resistance genes and may exhibit opportunistic pathogenic behavior under certain conditions (Guevarra et al. 2021). Horizontal gene transfer among enterobacteria on plant surfaces may contribute to the spread of antimicrobial resistance (Yin et al. 2022). The application of DFEO combined with vacuum packaging in the present study resulted in a reduction of coliform bacteria by 1.26 log CFU/g, indicating a significant inhibitory effect on the microbial population.

Observed shifts in microbial populations suggest that DFEO exerts selective pressure on susceptible taxa. Similar ecological dynamics have been described in studies examining the impact of essential oils on microbial communities associated with fresh produce (Gurtler and Garner 2022; György et al. 2020). Vacuum packaging alone provided partial microbial control by reducing oxygen availability and modifying atmospheric composition. Consistent with the significant main effect of treatment from two‐way ANOVA, the combination of DFEO with vacuum packaging produced a stronger inhibitory effect than either treatment alone, demonstrating the enhanced antimicrobial performance of combining chemical and physical preservation strategies. Whether this represents true synergy would require formal interaction analysis. Similar combined inhibitory effects between essential oils and modified atmosphere conditions have been reported by Hyun et al. (2015). Similarly, Gurtler and Garner (2022) reviewed the antimicrobial efficacy of several essential oils applied to fresh leafy vegetables and reported that the combination of essential oil treatment with modified atmosphere or vacuum packaging consistently produced stronger microbial reduction than either treatment alone. György et al. (2020) demonstrated that essential oils from thyme and oregano reduced TVCs on fresh vegetables by 1–2 log CFU/g when combined with physical preservation strategies, which is consistent with the reduction of 1.26 log CFU/g observed in the present study for coliform bacteria.

### 4.5 Insecticidal Activity and Potential Applications

The insecticidal assays demonstrated strong fumigant activity of DFEO against both *C. maculatus* and *M. dorsalis*. Although stored‐product beetles are not typical spoilage organisms of fresh leafy vegetables, the inclusion of insecticidal testing in this study was justified by the multifunctional character of DFEO as a natural bioactive agent. The volatile components of DFEO, particularly linalool and limonene, are known to exert both antimicrobial and insecticidal effects through related mechanisms involving disruption of membrane integrity and interference with nervous system function. Evaluating both activities within a single study contributes to a comprehensive characterization of the biological potential of DFEO for food protection applications. The LC_50_ values obtained in this study (18.09% for *C. maculatus* and 20.85% for *M. dorsalis*) are comparable with those reported by Moura et al. (2023) for linalool‐rich essential oils against storage pests. Similar LC_50_ ranges were also reported by Gupta et al. (2023) for *Lavandula angustifolia* essential oil containing comparable levels of linalool.

The similar susceptibility of both insect species suggests that DFEO may target conserved physiological pathways in storage pests. Previous studies have proposed that monoterpene alcohols may affect the nervous system through interactions with cholinergic or octopaminergic receptors (Liu et al. 2022; Ocampo et al. 2023).

The fumigant activity observed in this study represents a significant advantage for storage pest control, as volatile compounds can penetrate areas that are difficult to reach using conventional contact insecticides. Similar observations were reported by Karabörklü and Ayvaz (2023), who demonstrated efficient dispersion of monoterpene vapors in enclosed storage environments. The high volatility of linalool and limonene facilitates distribution of active compounds in the air, allowing effective fumigation at relatively low concentrations (Liang et al. 2022).

It should be noted that the insecticidal assay was conducted as a preliminary fumigant bioactivity screening under closed laboratory conditions using a wide concentration range including 100% essential oil. The practical applicability, safety, and feasibility of applying such concentrations in food or storage environments require further investigation, and the present results should be regarded as preliminary bioactivity data rather than evidence of practical applicability.

### 4.6 Technological Implications

The combination of antimicrobial, preservative, and insecticidal properties observed for DFEO highlights its potential as a multifunctional natural agent for food protection. The combined inhibitory effects observed between DFEO and vacuum packaging suggest opportunities for developing combined preservation strategies and active packaging systems incorporating natural volatile compounds (Perumal et al. 2022). Based on the results obtained, DFEO should be regarded as a complementary rather than alternative antimicrobial agent, intended to be used in combination with existing physical preservation strategies such as vacuum packaging or modified atmosphere, rather than as a standalone replacement for conventional antimicrobial treatments.

The consistent biological activity observed across the antimicrobial and storage experiments in this study further supports the feasibility of practical applications, although direct stability testing of the chemical profile was not performed, as noted above. Similar multifunctional applications of terpene‐rich essential oils have been reported by Rout et al. (2022).

Future research should focus on optimization of formulation strategies, evaluation of long‐term stability of bioactive components, and assessment of sensory acceptability of treated food products. In addition, combining essential oils with other natural antimicrobial compounds may further enhance preservation efficiency while maintaining product quality (Basavegowda and Baek 2021).

However, the potential impact of DFEO on the sensory properties of treated products represents an important limitation of the present study that should be addressed in future research. Linalool‐rich essential oils are characterized by a pronounced floral aroma, which may be perceptible in treated food products even at low application concentrations and may consequently influence consumer acceptance (Gómez‐Llorente et al. 2024). Future studies should therefore include standardized sensory evaluation panels to assess the acceptability of DFEO‐treated fresh produce, and optimization of application doses to balance antimicrobial efficacy with sensory quality.

## 5 Conclusion

The comprehensive evaluation of the tested batch of DFEO (*C. aurantium* var. *daidai*) confirmed its potential as a multifunctional natural compound with antimicrobial, preservative, and insecticidal properties, though these findings may not necessarily reflect the general performance of DFEO from different origins, harvest seasons, or extraction methods. GC–MS analysis identified a neroli‐type chemotype dominated by linalool, limonene, and linalyl acetate, which explains the observed broad‐spectrum biological activities. Antimicrobial testing demonstrated pronounced effects against both Gram‐positive and Gram‐negative bacteria as well as yeasts, with Gram‐positive bacteria showing the highest susceptibility. In situ experiments confirmed the practical applicability of DFEO under conditions simulating real food systems, with strong concentration‐dependent antimicrobial activity across all tested food matrices. The storage experiment on arugula demonstrated that the combination of DFEO with vacuum packaging represents the most effective strategy for improving the microbiological quality of fresh leafy vegetables during refrigerated storage. MALDI‐TOF MS identification revealed a complex culture‐dependent bacterial isolate profile associated with arugula, dominated by members of the family Pseudomonadaceae. Preliminary insecticidal screening against *C. maculatus* and *M. dorsalis* indicated fumigant activity at higher concentrations, warranting further investigation of its practical applicability before conclusions can be drawn regarding real‐world use. The obtained results, based on the specific tested DFEO batch, the arugula sample used, and the measured microbiological indicators (coliform bacteria and TVCs), support the potential of DFEO for the development of sustainable technologies for food protection, without extending to broader claims of shelf‐life extension or sensory quality. Future research should focus on the optimization of formulations for commercial applications, evaluation of the long‐term stability of bioactive compounds, and assessment of the sensory acceptability of treated food products.

## Author Contributions

**Minhang Qiao**: formal analysis, writing – review and editing. **Joel Horacio Elizondo‐Luevano**: formal analysis, writing – review and editing. **Anis Ben Hsouna**: formal analysis, writing – review and editing. **Rania Ben Saad**: formal analysis, writing – review and editing. **Zhaojun Ban**: formal analysis, writing – review and editing. **Li Li**: writing – review and editing, formal analysis. **Jian Lou**: formal analysis, writing – review and editing. **Stefania Garzoli**: methodology, data curation, investigation, formal analysis, writing – review and editing. **Miroslava Kačániová**: methodology, investigation, writing – original draft, formal analysis, supervision, project administration, visualization, funding acquisition.

## Funding

This research was funded by the grant APVV‐20‐0058 “The potential of the essential oils from aromatic plants for medical use and food preservation” and by the VEGA grant 1/0059/24 titled “Chemical properties and biological activity (in vitro, in vivo and in situ) of plant volatile mixtures, their main components and inclusion systems.”

## Conflicts of Interest

The authors declare no conflicts of interest.

## Data Availability

The authors have nothing to report.
